# Wireless Sensor Networks for Ambient Assisted Living

**DOI:** 10.3390/s131216384

**Published:** 2013-11-29

**Authors:** Raúl Aquino-Santos, Diego Martinez-Castro, Arthur Edwards-Block, Andrés Felipe Murillo-Piedrahita

**Affiliations:** 1 College of Telematics, University of Colima, Avenida Universidad 333, C. P. 28045 Colima, Col., Mexico; E-Mail: arted@ucol.mx; 2 Department of Automation and Electronics, Autonomous University of the West, C11 25# 115-85 km. 2 vía Cali-Jamundí, Colombia; E-Mails: dmartinez@uao.edu.co (D.M.-C.); afmurillo@uao.edu.co (A.F.M.-P.)

**Keywords:** wireless sensor networks, Ambient Assisted Living, arrhythmia detection algorithm

## Abstract

This paper introduces wireless sensor networks for Ambient Assisted Living as a proof of concept. Our workgroup has developed an arrhythmia detection algorithm that we evaluate in a closed space using a wireless sensor network to relay the information collected to where the information can be registered, monitored and analyzed to support medical decisions by healthcare providers. The prototype we developed is then evaluated using the TelosB platform. The proposed architecture considers very specific restrictions regarding the use of wireless sensor networks in clinical situations. The seamless integration of the system architecture enables both mobile node and network configuration, thus providing the versatile and robust characteristics necessary for real-time applications in medical situations. Likewise, this system architecture efficiently permits the different components of our proposed platform to interact efficiently within the parameters of this study.

## 1. Introduction

Advances in wireless, sensor design and energy storage technologies have contributed significantly to the expanded use of Wireless Sensor Networks (WSN) in a variety of applications. Integrated micro-sensors with onboard processing and wireless data transfer capability, the most important components of WSNs, have already existed for some time [1,2]. However, at present, more efficient designs have successfully integrated a wide range of sensors. These sensors can monitor a large variety of environmental factors that can affect health including temperature, humidity, barometric pressure, light intensity, tilt, vibration and magnetic field intensity among others, using short-distance wireless communications.

The enormous cost of providing health care to patients with chronic conditions requires new strategies to more efficiently provide monitoring and support in a remote, distributed, and noninvasive manner. Diverse European projects such as the “HealtService24 Project” are trying to improve the quality of medical attention by providing remote medical monitoring. These types of projects are currently developing mobile monitoring systems and integrating remote monitoring into their healthcare protocols to provide expanded healthcare services for persons who require monitoring and follow-up, but do not require immediate medical intervention or hospitalization.

The importance of monitoring patient health is significant in terms of prevention, particularly if the human and economic costs of early detection can improve patient independence, improve quality of life, and reduce suffering and medical costs. The early diagnosis and treatment of a variety of diseases can radically alter healthcare alternatives or medical treatments. Prevention and effective control of chronic diseases has proven repeatedly to be more cost effective than conventional treatments at medical facilities. This is particularly true with chronic and incapacitating illnesses such as cardiovascular disease or diabetes. In the case of cardiovascular disease, 4% of the population over 60 and more than 9% of persons over 80 years of age have arrhythmias, or abnormal heart rates, which require occasional diminutive electrical shocks applied to the heart. Sensors can identify at-risk patients by monitoring and transmitting their real-time cardiac rhythms to medical professionals who can subsequently determine whether or not they require a pacemaker to assist establish and maintain normal sinus rhythm [3].

Body sensor networks used to manage diabetes will one day involve implanted sensors, not only to monitor patient glucose levels, but also to administer insulin in a timely fashion. In sum, the abovementioned chronic diseases exemplify the need for biochemical and physiological continuous monitoring.

## 2. Related Works

The continuous monitoring and analysis of vital signs is the key to detecting potential health risks in otherwise healthy-looking patients. There are presently several projects around the world whose goal is to monitor the patient health. The authors in [4] describe the BASUMA project, which focuses on developing a robust and energy efficient platform for human wireless body sensor networks to provide at-home monitoring of chronically ill patients. The initial goals of the BASUMA project are: to improve the treatment of obstructive pulmonary disease and provide support for female breast cancer patients undergoing chemotherapy. In [5], the author describes how to implement a personal sensor network to monitor patients and help provide health care. This project combines several intelligent sensors and an integrated control node that functions in conjunction with a Bluetooth network. In [6], the authors present a system based on wireless sensor network technology. This project describes an architecture composed of medical sensors incorporated around the human body using the ZigBee standard. The WHAM-Bios project in [7] proposes telemedicine applications to provide real-time emergency medical services. The WHAM-Bios project is based on a device the authors call “Human Body Gateway,” where the sensor nodes provide the information needed to produce instantaneous monitoring results. Real-time monitoring requires algorithms that facilitate contention-free communication in order to reduce the power needed to transmit data.

The long-term health effects of the electromagnetic fields caused by in-home monitoring has been a cause of concern. The author in [8], focuses on the safety of body sensor networks and wireless communications in close and constant proximity to humans.

In [9,10], the objective is to incorporate technologies into clothes or common accessories (for example, watches, bracelets, *etc*.) to measure, register and transmit different physiological parameters, including: heart rate, body temperature and movement. The authors in [11] describe a prototype that monitors diabetic patients and the authors in [12] describe another prototype of a retinal prosthesis, based on embedded implanted intelligent sensors.

There are various projects that use 802.15.4 and ZigBee to transfer patient information. The authors in [13] present ZUPS. This is an ultrasound based position system that provides multi-cell coverage. The system uses ZigBee and ultrasound to measure distances between mobile devices carrying tags and beacons with known locations; however, it uses proximity and multi-lateration localization methods simultaneously. This combination reduces the infrastructure needs for the ultrasound system and also provides accurate information even in very short distances; this enables the system to provide guidance and spatial orientation training inside buildings for the elderly and people with disabilities.

Projects presenting plural layer architecture have also been developed. In [14] a three-layer network structure for a pervasive medical supervision system is proposed. The first layer is the medical sensor network that provides oximetry, heart rate and blood pressure information, as well as contextual data such as temperature and the patient's video/picture in emergency situations. This network is configured in a star topology with a gateway node. The second layer provides a reliable transmission stream that permits data to be transmitted to the nearest wireless node located in the house. The transmission can then be relayed to a PC with an Internet connection inside the house. The third layer of the system is responsible for the compiling physiological data in a remote medical center for analysis to provide feedback to the patient by means of a standard mobile phone, PDA or web services.

Some projects developed aim to significantly reduce long-term network power consumption for monitoring applications where there is no need to alert the patient or health care provider about a threatening event. SATIRE [15] is designed to identify user activity based on accelerometer and global positioning system (GPS) readings. The system uses SHIMMER motes and accelerometers to sense and record data to a local memory mechanism. These data are then opportunely transmitted using a low-power radio device when the SHIMMER node is within communication range of the base station, thus extending the duty cycle of the node by increasing battery life. The data is then processed offline to characterize the user's activity patterns.

In the medical application field, there have been some projects focusing directly on ECG measurement and data processing. A variable control system is proposed in [16] to optimize the measurement resolution of ECG readings to save power. This system allows users to set the ECG Signal-to-Noise-Ratio, thus permitting them to select the exact resolution to meet their needs. It is important to note that the higher the resolution selected, the greater the amount of energy required to save and transmit information. Therefore, by selecting the exact resolution, users avoid registering and transmitting more information than is actually required by the health care professional.

CodeBlue [17] is a hardware and software platform developed at Harvard University. The network architecture is based on the publish/subscribe routing framework. The sensors do not publish data at an arbitrary rate because the platform filters the data locally. A multi-hop routing protocol can be used when the subscribers and publishers are not within a single hop radio range. The publishers and subscribers are mobile, so position information has to be available to define routing paths. This information is obtained using a localization system called MoteTrack [18].

With the increasing popularity of mobile ECG measurement, data transmission to a remote place for processing and diagnosis (such as a medical center) gains importance. In [19] the authors describe a cardiac healthcare system that can use WLAN and CDMA technologies to transmit data. When the ECG sensor detects a WLAN, it transmits data using that path; otherwise, a cell phone with a prototype wireless dongle performs a simple electrocardiogram diagnosis algorithm. Again, in order to save energy, the data is transmitted only when an abnormality is detected.

Mobile ECGs [20] measure and analyze the user heart rates by means of a smart mobile phone that functions as a base station. The ECG is then transmitted via Bluetooth back to the mobile phone for the patient to view and take the appropriate actions. However, if an abnormality is discovered, the mobile phone analyzes the received data and sends the ECG data to a server for further analysis by healthcare professionals.

## 3. Medical Sensor Applications for Closed Spaces

Currently, cardiovascular problems represent a major cause of death in the entire world. The great interest in developing clinical devices to detect and continuously monitor cardiovascular diseases is somewhat limited, as transient abnormalities cannot always be monitored. However, many of the diseases associated with the cardiovascular system are related precisely to transient episodes rather than continuous abnormalities, such as transient surges in blood pressure, arrhythmias, *etc*. These abnormalities cannot be predicted because their analyses, even through stress tests, often do not detect them in a reliable and timely manner using conventional protocols. Therefore, these events must be monitored under actual living conditions to diagnose some heart conditions. The result of this approach is to improve the patient's quality of life and change patient behavior patterns, resulting in a reduction of therapy costs.

### 3.1. Arrhythmia Detection Algorithm

The goal of this study is to take ECG measurements and detect arrhythmias of subjects who are in motion during a rehabilitation session in an enclosed 100 m × 100 m space, which represents the dimensions of typical clinics and rehabilitation centers. The sampling period chosen is based on the signal bandwidth of the electrocardiogram (ECG), which, according to the American Heart Association, has harmonics from 0 to 100 Hz. The most relevant information used to monitor arrhythmias, however, is between 0.5 Hz and 50 Hz.

The ECG frequency spectrum can be established with the relevant components of the signal (QRS complex and waves P and T) between 0 and 35 Hz. The Nyquist sampling theorem provides a minimum sampling period (*T_s_*) of about 14 ms. However, for practical purposes, it is necessary to use a sampling period of [*T_s_*/8, *T_s_*/4], so we selected a 3 ms interval.

Arrhythmia detection algorithms rely mainly on the detection of the ECG QRS complex. Currently, there are various algorithms that detect this complex. For example, there are algorithms based on neural networks [21], amplitude of first and second derivative algorithms [22], genetic algorithms [23], algorithms that use the wavelet [24], hybrid algorithms [25], bank filter algorithms [26], algorithms based on the correlation of the signal with sample beats [27], and heuristic algorithms based mainly on non-linear transforms [28].

Wireless sensor networks employ nodes that possess limited resources. Consequently, the case study analyzed in this work exclusively considers monitoring ECG signals in real-time conditions. Because of this, it was important to select a QRS detection algorithm that has the necessary sensibility and precision, but most efficiently employs limited resources such as energy, memory and the CPU, without using too many resources. Some algorithms that rely on the Wavelet and Hilbert transform perform well, possessing adequate sensibility and precision, but employ a lot of resources [29]. For this reason, we selected the Pan and Tompkins algorithm because it has relatively simple filters, non-linear transformations and decision methods. Importantly, however, the Pan and Tompkins algorithm is sufficiently precise and possesses adequate sensibility values [30].

**Figure 1.**
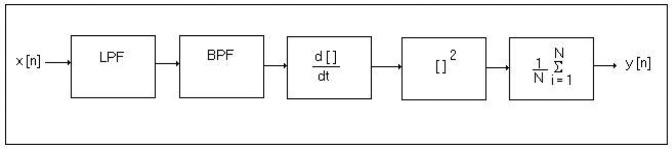
Block diagram of the Pan and Tompkins algorithm.

This algorithm employs a band pass filter that sets a low pass filter (LPF) and a high pass filter (HPF) to reduce noise, which serves to provide interference against signals outside the frequency band in which the QRS operates. To complement this process, the Pan and Tompkins algorithm has a derivative function that emphasizes the slopes of the R wave. Then, a quadratic function is applied to the resulting signal of the derivative function to further enhance the high frequency characteristics of the QRS complex. Finally, the energy estimate is made with a mobile window size of the longer QRS complex. Figure 1 provides a block diagram of the Pan and Tompkins algorithm with the steps involved in analyzing the ECG signal.

**Figure 2.**
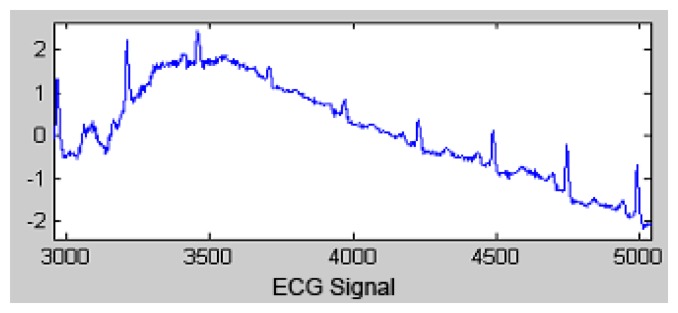
ECG vector with noise.

Figure 2 shows how the Pan and Tompkins algorithm functions. Initially, an ECG wave with noise is presented.

The transfer function low-pass filter is:

(1) $$H(z)=\frac{{\left(1-z^{-6}\right)}^{2}}{{\left(1-z^{-1}\right)}^{2}}$$

**Figure 3.**
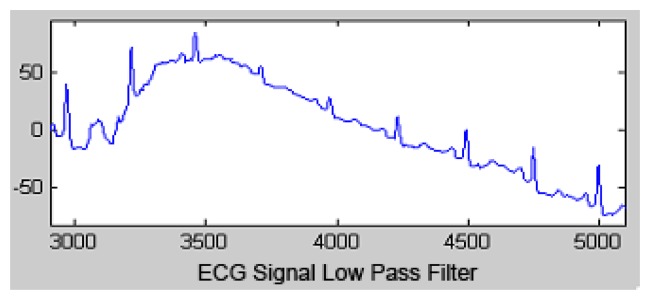
Low pass filtered signal.

The output signal of the LPF is shown in Figure 3.

The transfer function of high pass filter is:

(2) $$H_{hp}(z)=\frac{P(z)}{X(z)}=z^{-16}-\frac{H_{lp}(z)}{32}$$

**Figure 4.**
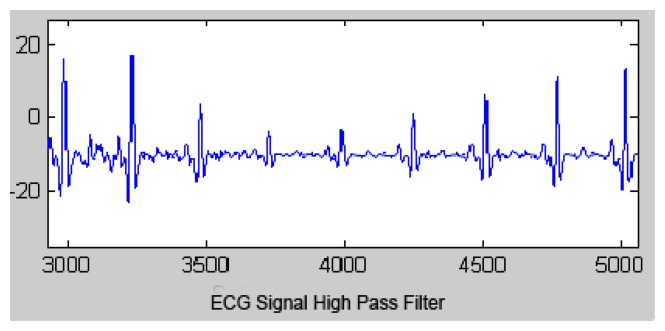
High pass filtered signal.

The output signal of the HPF is shown in Figure 4.

After the signal is filtered, most of its energy is contained in the QRS complex. Then, the derivative function is applied, high frequencies are accentuated and the low frequencies are attenuated. Therefore, the high slopes are highlighted to generally distinguish the QRS complex within the ECG signal.

The transfer function of the derivative function is:

(3) $$H\left(z\right)=0.1\left(2+z^{-1}-z^{-3}-2z^{-4}\right)$$

**Figure 5.**
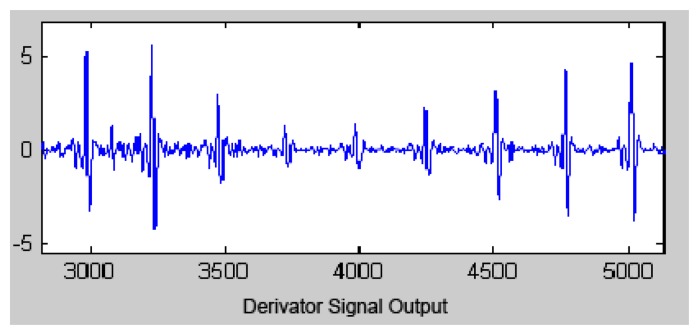
The derivative function output signal.

The output signal of the derivative function is presented in Figure 5.

**Figure 6.**
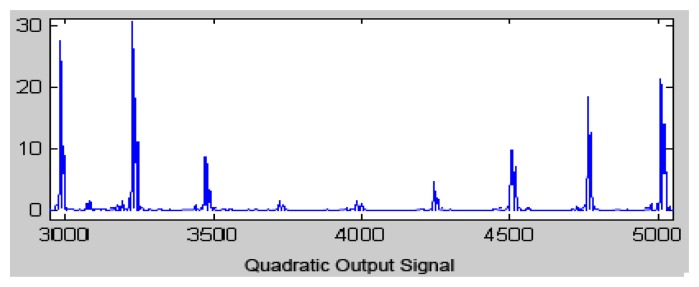
Quadratic output signal.

Previously, to carry out the window integration process, the quadratic function was applied for all signal points to be converted into positive values that emphasize the high frequency signal, which is mainly the QRS complex (Figure 6).

The equation that implements this operation is:

(4) $$y(nT)={\left[x(nT)\right]}^{2}$$

The sampling window size (*n*) should always be greater than or equal to the largest duration QRS, but if the window is too long, the integration could add information to the T wave. On the other hand, if the window side is too short, the T wave will not be sufficiently amplified, possibly resulting in erroneous peak detection.

**Figure 7.**
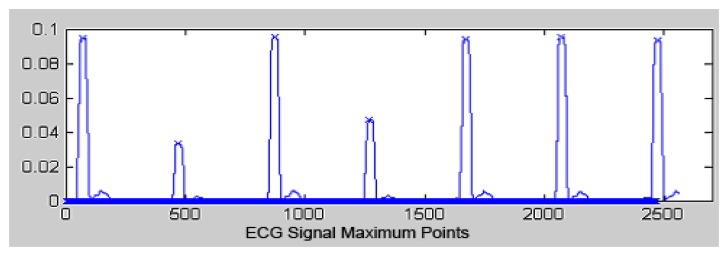
Maximum points.

A peak detection algorithm is then applied to the Pan and Tompkins algorithm to identify the precise instants that ECG wave segments occur (Figure 7). Subsequently, a separation time analysis is realized between two R segments. The QRS complex duration and the R wave height are used to detect the presence of arrhythmias [21]. This algorithm consists of:

- Seeking the initial point in which the filtered signal exceeds the detection threshold.
- Calculating the absolute maximum window size of the filtered signal.
- Determining the R point of the ECG signal.
- Actualizing the hop.
- Updating the threshold.

### 3.2. Architecture Node

**Figure 8.**
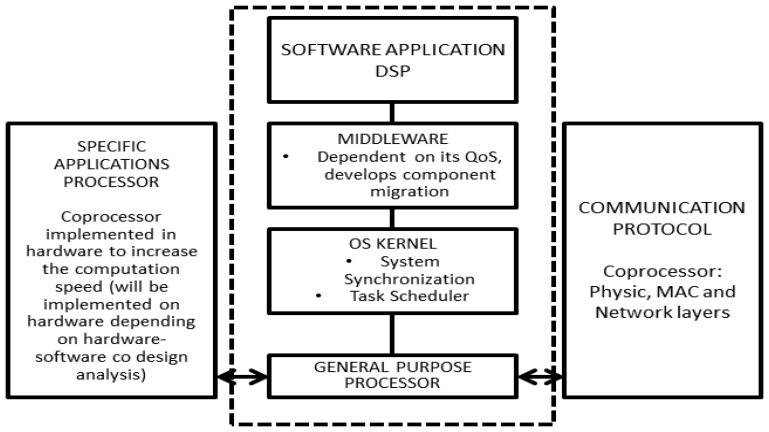
Node architecture.

The proposed generic architecture for nodes in wireless sensor networks for point-of-care diagnosis is presented in Figure 8. Some important points to mention are:

- The architecture enables co-design of the hardware and software components. This feature allows users to optimize and develop the distributed components required for their implementation in hardware and software applications, thereby obtaining a balance between cost, power consumption and processing time.
- Communication between the nodes and local coordinators is done through wireless networks.
- The kernel uses a scheduler, which allows users to meet application deadlines and optimize power consumption.
- It updates its QoS indices. The architecture uses these indices and those of its neighbors to request another node in its sub-network to migrate, create or destroy application components (some of which are, in fact, exact copies of others).

### 3.3. Analysis of Computational Requirements

We compared the performance of the arrhythmia detection algorithm to select a set of processor architectures that would perform adequately. Presented in the previous section, the four types of processors currently used for deploying nodes in sensor networks were used: ARM7TDMI, MSP430, PIC18, and MC9S08GB60.

For the analysis, the same operating speed of 8 MIPS was used for each processor. The total computation time of the implementation of the Pan and Tompkins algorithm is presented in Table 1. The total computation time was estimated using the sum of the individual function values of each architecture (derivation, squaring and integrating window). Results show that the ARM architecture requires a lower utilization rate (U), while the PIC architecture requires a higher utilization rate (U).

Another aspect considered is the active mode power consumption (PA) of the four architectures during the execution of the Pan and Tomkins algorithm, which is related to the four architectures' power consumption in active mode with their respective percentage use of CPU capacity.

Table 2 shows the PA × U parameter. The ARM7 architecture has a value very close to the MC9S08GB60 architecture. Therefore, these two architectures are suitable for use in this study. The MSP430 architecture has the lowest value, making it the best of the four architectures insofar as the PA × U parameter is concerned.

## 4. Network Performance Analyses

### 4.1. Network Architecture for Confined Space Healthcare Applications

**Figure 9.**
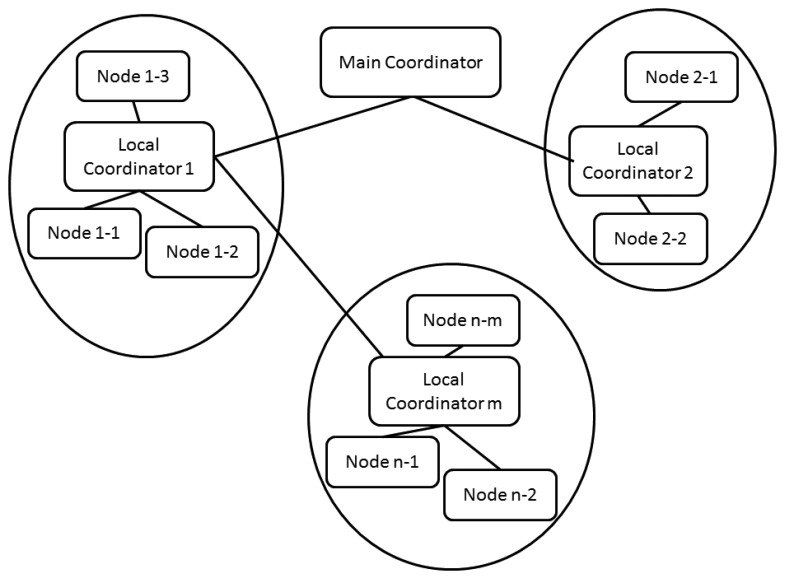
Network architecture.

Figure 9 presents a proposed generic network architecture that integrates different types of nodes. It should be noted that the approach includes a plan that provides increased integration between the different architecture levels of the application. This is convenient because the architecture can make the optimal decisions necessary to reconfigure the system to provide improved QoS. The general goals of the architecture are to:

- Minimize latencies.
- Optimize power consumption.

The main coordinator node is responsible for coordinating the complete application. It has a fixed location and communication with local coordinators is supported by means of wireless links. It sends synchronization signals to the local coordinators of the sub-networks.

The Local Coordinator controls the activity inside the sub-network and carries out the following functions:

- Transmits information from a single mobile node located in each patient.
- Sends synchronization signals to nodes in its sub-network which sample variables for patient's state analysis
- Develops routing packets between sub-networks using multi-hop techniques.
- Distributes QoS indexes of nodes, which belong to its sub-network.
- Calculates its sub-network QoS index, and distributes this value and its neighboring sub-networks indexes (those reached in a single communication hop) between nodes in its sub-network, where it can either:
  ○ Accept linking new nodes to sub-network.
  ○ Update the best routes in the routing tables of data (which will be function of hops and the utilization percentage -information transmitting- of each router node).

### 4.2. Case Study

As a first approach to determine the optimal architecture to use in this study, we undertook a performance analysis of a study employing the IEEE 802.15.4 standard. The considerations we consider in our proposed solution include:

- Transmitting nodes send packets from the patient node to a sink node every 3 s; the data frame consists of 2 Bytes that contain each patient's code and the type of arrhythmia detected.
- Receiving nodes wait for an acknowledgment packet (ACK) after each transmission. If a receiving node does not receive a response in less than 100 ms, the transmitting node re-sends the information packet. If after 25 attempts, the receiving node does not obtain any response, this node changes its status to an error node.

**Figure 10.**
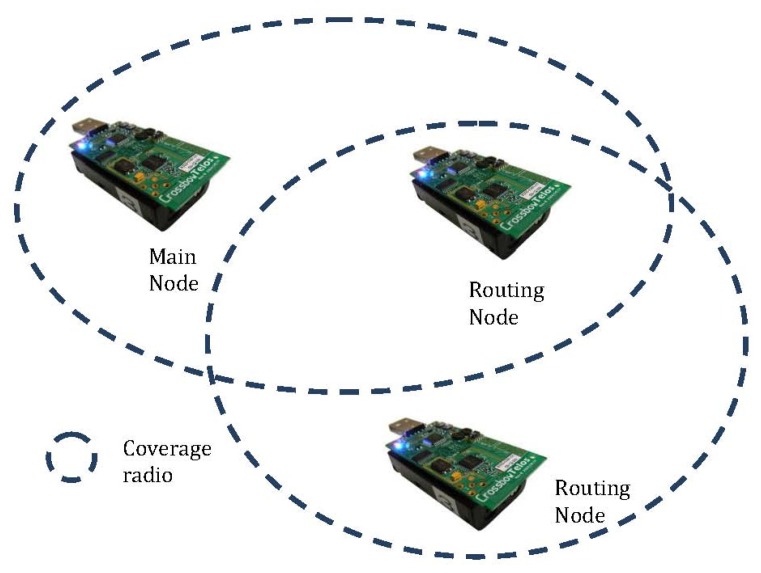
Node distribution and coverage in the study.

The node distribution is shown in Figure 10. This distribution allows us to determine the possible patient locations, taking into account the specifications of the selected device for implementing the physical layer, CC2420, whose characteristics include:

- A coverage radius of 30 m and 100 m without obstacles.
- A frequency range of 0.4–2.4835 GHz.
- Data rates of 250 Kbps.

**Figure 11.**
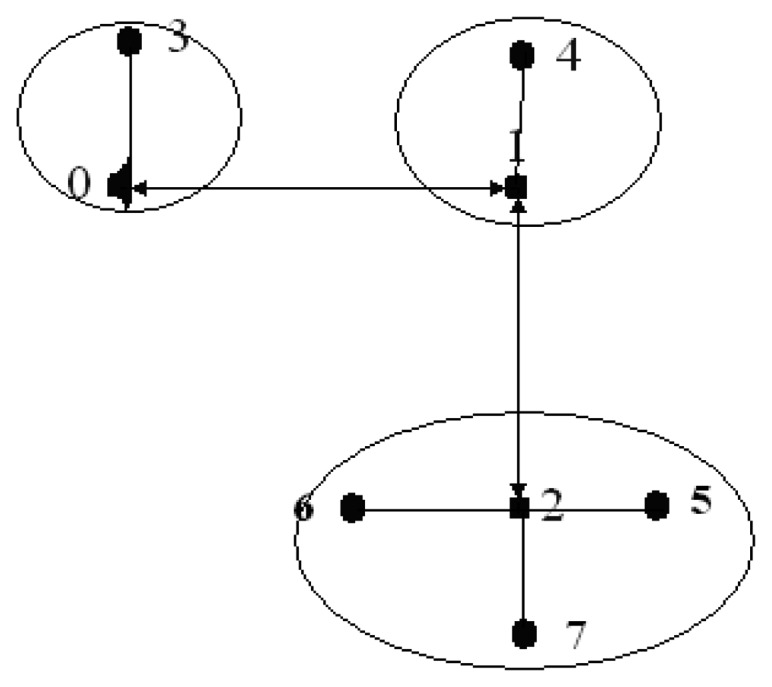
Global network of this study.

We propose implementing a network as presented in Figure 11, which consists of 3 fixed nodes that will not have power consumption restrictions. These three nodes will receive the reports transmitted by the sensors located on five patients that route the message to the sink node. Fixed nodes have identifiers 0, 1 and 2 and the sink node has the ID 0. Patient devices have IDs from 3 to 7.

The routing network consists of nodes 1, 2 and node 0 (network coordinator). Each one of these nodes forms a sub-network in collaboration with patient nodes. Because of their mobility, nodes enter and exit the sub-network continuously changing the configuration and network structures.

The simulation for this study was conducted in TOSSIM. Additionally, the TelosB platform was selected because it includes the CC2420 transceiver and the MSP430 processor. Given the nature of this study, where the network backbone nodes are fixed, a routing algorithm that uses a fixed table (see Table 3) was used.

This study simulates a critical case where all the mobile nodes are connected to the most distant sub-network from the sink node. The transmission times for 2 Bytes from all the patients to the main node are presented in Table 4.

Data1 means that the message transmission from a mobile node to the main node has terminated. Data2 means that the corresponding node has not received the ACK message.

## 5. Experimental Results

The following section presents experimental results of a network topology used for biomedical applications. We realized a performance analysis using a star topology that only considered one of the routers in the global topology, which had three end devices connected to it.

### 5.1. Network Configuration

The network consisted of three End Devices and one Router. The first end device (named ECG) transmitted a 320-Byte ECG signal. This signal was transmitted using a frame, which had a 20-Byte protocol field and an 80-Byte payload size. The frame size was specifically chosen to send the ECG signal in the minimum amount of frames possible. The other two devices transmitted temperature information in a single frame. In all cases, in order to simplify transmissions, we used a 100-Byte MSDU with no security configurations, using short addresses and ACK requests. All the transmissions were performed on Channel 21 of the IEEE 802.15.4 standard.

The ECG signal was transmitted every thirty seconds and was transmitted in the FCAP region of the super frame structure. The other devices transmitted their information every twenty seconds in the CAP region.

Two types of tests were realized. The first was a set-up time test. In this test, the time that all the network devices needed to join a network was measured. In the second test, the percentage of packets arriving at their destiny on the first attempt was counted.

### 5.2. Hardware Used

The tests were realized using the TelosB [31] and SHIMMER2R platforms. The TelosB and SHIMMER2R employ the CC2420 chip radio and the MSP430F11 processor. Importantly, the CC2420 implements the PHY layer of the IEEE 802.15.4

The TelosB were used as the network devices while The SHIMMER2R acted as the coordinator. Finally, a Texas Instruments CC2531 USB dongle (Texas Instruments CC2531 USB Dongle [32]; was used as a sniffer tool. This dongle uses the CC2531 radio chip and can receive IEEE 802.15.4 frames.

### 5.3. Software Used

The platforms mentioned above employed the most updated version of TinysOs 2.x (trunk 5535). Additionally, all the platforms used the MAC layer implementation established by the IEEE 802.15.4 standard. This implementation was developed by researchers from the CISTER Research Unit in synergy with the TinyOS 15.4 and ZigBee working groups. We performed some modifications regarding ACK handling, transmission and reception of PANIDs. The Texas Instruments SmartRF Packet Sniffer version 2.13.63.0 was used for packet analysis.

#### 5.3.1. Main Node State Diagram

**Figure 12.**
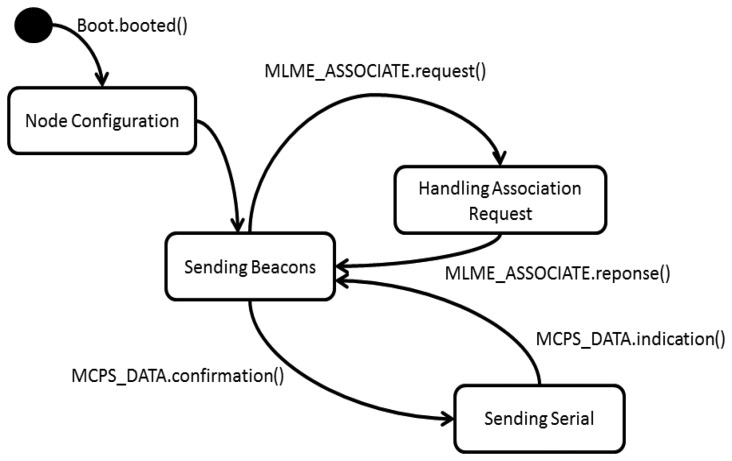
Main Node State Diagram.

According to the system requirements and the tools provided by the IEEE 802.15.4 standard, the following State Diagram for the Main Node was designed (Figure 12).

The main node starts operating with the Boot.booted event; at that point, it begins its configuration process and starts the beacons transmission. When it receives an association request (MLME_ASSOCIATE.request), the node attends the request and sends an answer to the request transmitter node. Then, when receiving information packages from the associated nodes, it retransmits them through the serial port to a computer.

#### 5.3.2. Network Device State Diagram (Figure 13)

The network device starts its configuration when the event Boot.booted in TinyOS is reported. When receiving a beacon, it transmits an associating request to the Coordinator. When the Coordinator answers the request, two processes initiate:

(a) The ADC is configured to do a periodic selection of 333 ECG samples.
(b) A periodic Timer is configured every 30 s for signal transmission (ecgTimer).

When the signal sampling ends (ecgDataready) the Pan Tompkins algorithm starts, this algorithm was implemented by posting tasks for each processing phase. If the algorithm detects an irregularity, it sets a flag to TRUE (troubleDetected) and when receiving the following beacon, it transfers the report and the original ECG signal. When the ecgTimer overflows, it sets a flag (dataReady) to do a routine transmission of the ECG signal at the next reception of a beacon.

#### 5.3.3. Application

The application was realized in TinyOS 2.1 with Crossbow TelosB development platforms. An application was created for the Coordinator and another one for the Network Device; therefore each platform could only perform one of the two behaviors. This alternative does not allow the established network to be an ad-hoc network, but it is a solution that notoriously reduces memory consumption in platforms.

There were three Network Devices, two of them transmitting the environment temperature information, by means of the SHT11x sensor integrated in the TelosB. The remaining Network Device made the implementation of the Pan Tompkins algorithm, described on Section 3.1. When the node completes its association process, a periodical sampling of the ECG signal is configured. When this signal is sampled, the Pan Tompkins algorithm initiates. Each algorithm block was developed in a different task and every corresponding task was posted at the end of the signal sampling.

This alternative was selected to allow synchronous events, such as the arriving of a beacon or data, could be attended without major delays. When concluding the filtering process, the Network Device transmitted the according information of each phase.

The network Coordinator received this information and transmitted it through the serial interface to the Computer.

### 5.4. Network Set-Up Time Tests

#### 5.4.1. Test Explanation

The objective of these tests was to obtain information about the network setup time. This time was defined as the time between the first beacon transmission and the last node's reception and its association response.

The test was repeated five times for every configuration and all the nodes were turned on concurrently. At the end of the tests, the data related to each configuration was averaged.

A star topology was used in all occasions and the nodes were located on the chest, waist and ankle of a subject's clothing. The subject was located 1 meter from the coordinator, which was connected to a PC. Two AA batteries powered the devices.

Two types of tests were performed. Only one star was active in the first test and there was no additional activity in the channel. In the second test, two stars actively worked in the same channel, but with different PANIDs.

Each time measurement was performed with beacon order (BO) values of 7 and 9. For each of these values the duty cycle values were set at 0.25, 5 and 1. The duty cycle is the relation between the standard deviation (SD) and BO. The transmitted frames were analyzed using the Texas Instruments Sniffer and a CC2531USB Dongle, which was 15 cm distant from the coordinator.

#### 5.4.2. Results

Table 5 shows the average network set-up time with a star working in Channel 21 and a beacon order of 9. Table 6 shows the standard deviation of the data that was operated to obtain each average time.

The data in Tables 5 and 6 show that varying the super frame duty cycle does not significantly affect the average set-up time. However, there are significant variations regarding the standard deviation.

With two stars working, a modification in the duty cycle generates variations in the average set-up time, which can be explained by the traffic increase in that channel. This, in turn, creates greater delays in the transition and reception of MLME-ASSOCIATE primitives. The longest set-up time observed was when the duty cycle was 100%.

Table 7 shows the average set-up time with one star in the channel and a BO of 7. Table 8 shows the standard deviation of the data handled to obtain the average times of Table 3.

When the duty cycle varies in a network with a BO of 7, there are greater differences in the average set-up time when compared with results in a network with a BO of 9. This is a consequence of, on some occasions, the reduced time available to transmit the association primitives. This causes some nodes to have to wait for the next beacon to successfully end their association process. This time variation was present in both one- and two-star network configurations.

### 5.5. Retransmission Tests

#### 5.5.1. Test Explanation

These tests aimed to measure the percentage of frames that are successfully received on the first attempt and what percentage needed to be retransmitted. To accomplish this, 500 packets were transmitted with the ackRequest flag set to 1. Each device reported the result of its respective MCPS_DATA_CONFIRM primitive, which allowed us to check if the packet was acknowledged. Packets were transmitted at 30-s intervals.

The network used two types of network devices: “End Devices” and “ECG.” The first device transmitted a packet with a 100-Byte payload with information about temperature. The second device transmitted 160 samples of an ECG signal in four consecutively sent packets. Importantly, this transmission was performed in the FCAP region of the super frame structure.

In the case of duty cycle (D) = 0.25 and BO = 9, presented in Table 9, the end devices acknowledged 54.26% of the packets received by end devices in the first attempt; however, for the ECG, it was only 38.88%. This behavior can be explained by the short duration of the active section of the super frame, which is approximately 491 ms, as well as the temporal restrictions of TinyOS event handling and task posting.

ECG transmissions significantly improve when the duty cycle is set at 0.5. In fact, the efficiency increases to 60% in the first transmission. Importantly, however, the performance of the end device drops to 41.36%. It is possible that this is due to the priority given to processing the incoming data over the received ECG frames, which can cause delays in the ACK reply and an ackTimeOut. When the duty cycle is 1, the percentage of successfully transmissions in the first attempt increases to around 50% for both types of devices.

Table 10 shows the results of these tests for BO = 7. Table 10 shows a significant improvement in the performance of transmissions when D is 0.5 with a BO of 7, both in the case of end device and the ECG, with percentages of 57.70% and 67.57%, respectively. This performance even exceeds the configuration for the test results with a BO of 9. It is possible that with shorter beacon intervals the internal clocks of the nodes suffer shorter displacements, which improves their synchronization capacities with the beacons.

## 6. Security Concerns

One of the most important challenges for communications is how to ensure the patient security and privacy during transmission of data to avoid the threat from attacker [33]. The authors in [34] analyzed privacy threat types based on the wireless health monitoring system architecture and built the key system model for identity threat and context privacy preservation, based on traffic analysis threat. The authors mention that to resist these threats, the integrated message encryption, identity authentications and traffic context privacy preservation, based on identity-based cryptography (IBC) and identity-based signature (IBS), is carried out at one time during the process of sending, receiving and accessing the patients' health information. However, the authors in [35] argue that the results carried out for [34] lack context privacy and do not provide sufficient security for physicians. They propose a new platform they call the “u-healthcare system”, which provides security considerations for their future platform. In addition, authors in [35], mention that for Body Area Networks (BANs) to monitor and control a large variety of physical parameters in different contexts, it is necessary to tolerate a high degree of change and possibly even permit temporary privacy violations in order to meet functional safety or performance requirements. For example, an individual wearing an EKG might experience a heart arrhythmia and the real-time reporting of this incident takes precedence over already existing privacy requirements, such as is presented in our scenario.

## 7. Dynamic Routing Mechanism

Dynamic routing mechanism is another important issue in this work. Our proposal does not present a simulated or implemented routing mechanism for mobile ad hoc nodes, wireless sensor networks or vehicular ad hoc networks such as the PANDORA [36] or LORA-CBF [37,38] protocols.

The PANDORA protocol possesses two distinct layers: (1) An *ad hoc* network which is composed of Wireless Mesh Clients (WMC), and (2) Wireless Mesh Routers (WMRs) with a backbone connection between the WMRs. It is important to note that the two types of nodes of a Wireless Mesh Network (WMN) suffer different constraints. WMCs located at the end points have limited power resources and may be mobile, while WMRs possess minimum mobility, but do not suffer from power constraints.

LORA-CBF has been simulated and implemented in vehicular ad hoc networks and wireless sensor networks. It has also been implemented in a precision agriculture scenario. The implementation of either PANDORA or LORA-CBF will depend of the specific scenario. As we mentioned previously, PANDORA has been implemented for fixed and mobile nodes in home, urban and rural environments. On the other hand, LORA-CBF has been implemented using fixed nodes (agriculture) and highly mobile nodes (vehicles). The results simulated or implemented previously can be applied to this scenario in future work.

## 8. Conclusions

The proposed architecture considers very specific restrictions of applying sensor networks in clinical situations. The seamless integration among the different levels of the system architecture enables mobile node and network configuration. Likewise, this system architecture permits the interaction of its components to function efficiently within the parameters of this study.

Simulation results of this study indicate that:

- The routing algorithm based on fixed tables supported by the IEEE 802.15.4 standard functions well under the restrictions imposed by the case study.
- The MSP430 architecture performs well under the parameters described in this case study.
- Although the set-up time of IEEE 802.15.4 can vary significantly, it is small enough to be used to monitor medical applications.
- The presence of additional star topologies employing the same channel does not significantly affect the network set-up time.
- The performance of the transmissions in this case study is good; however, for critical applications, we suggest employing more energy saving techniques.
- The experimental results validate the proposed scenarios for medical applications in wireless sensor networks.

Future research will employ hardware we developed in conjunction with a hybrid protocol such as PANDORA for patient home monitoring.

## Figures and Tables

**Figure 13.**
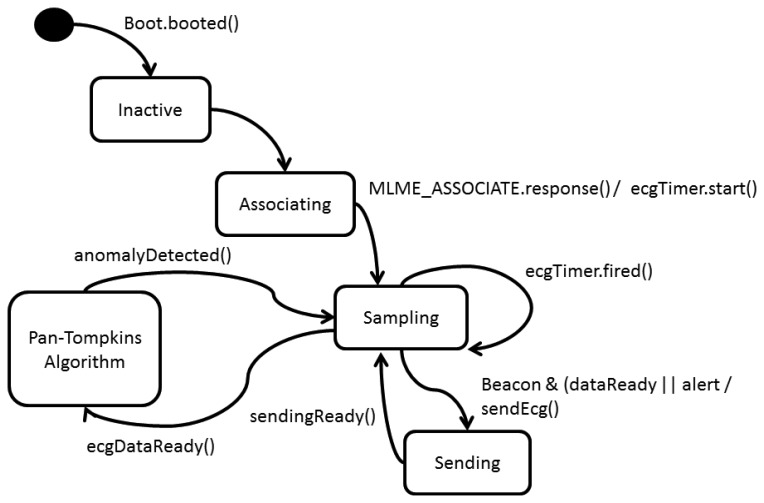
Network Device State Diagram.

**Table 1.** Computation time for the Pan and Tomkins algorithm.

**Processor**	**Derivation**	**Squaring**	**Integrating** **Window**	**Total** **Computing Time**	**Period** **[μs]**	**Utilization** **Rate (U)**
LPC2124–ARM	70.2 μs	142 μs	280.5 μs	492.7 μs	3,000	16.4%
MSP430F1611	191.9 μs	162.5 μs	697.8 μs	1,052.2 μs	3,000	35%
PIC18F458	406.2 μs	209 μs	1,083.7 μs	1,698.9 μs	3,000	56.6%
MC9S08GB60	497.2 μs	332 μs	707.35 μs	1,536.55 μs	3,000	51.3%

**Table 2.** PA × U parameters.

**Processor**	**Utilization Rate (U)**	**Active Power (P_A_) [mVA]**	**P_A_ × U**
LPC2124–ARM	16.4%	180	29.52
MSP430F1611	35%	19.2	6.72
PIC18F458	56.6%	220	124.52
MC9S08GB60	51.3%	51.6	26.47

**Table 3.** Routing table.

**Origen Node**	**Destination Node**
2	1
1	0
0	

**Table 4.** Transmission time for 2-Bytes from all the patients to the main node.

**Sender Node**	**Receiver Node**	**Time (s)**	**Source Node**	**Receiver Node**	**Time (s)**
6	2	78.309	**0**	**1(ack)_1_**–5 ends	0.613
4	2	0.320	7	2 Rtx_2_	0.684
7	2	0.333	3	2 Rtx_2_	0.684
**2**	**6(ack)**	0.344	**2**	**1 Rtx moving 4**_2_	0.684
5	2 Rtx_2_	0.355	**1**	**2 (ack)**	0.701
**2**	**1 (moving frame from 6)**	0.380	7	2 Rtx_2_	0.714
3	2 Rtx_2_	0.380	**1**	**0 (moving frame from 4)**	0.740
**1**	**2(ack)**	0.397	**2**	**7 (ack)**	0.746
**1**	**0 (moving frame from 6)**	0.421	**0**	**1 (ack)_1_**–4 ends	0.764
5	2 Rtx_2_	0.421	**2**	**1 (moving frame from 7)**	0.780
3	2 Rtx_2_	0.463	3	2 Rtx_2_	0.780
**2**	**1 (moving frame from 5)**	0.486	**1**	**2 (ack)**	0.792
7	2 Rtx_2_	0.486	3	2 Rtx_2_	0.807
**2**	**5(ack)**	0.488	**2**	**3 (ack)**_2_	0.825
**0**	**1(ack)_1_**–6 ends	0.488	**2**	**1 (moving frame from 3)**	0.860
**1**	**2(ack)**	0.500	**2**	**1 Rtx moving 3**_2_	0.877
4	2 Rtx_2_	0.513	**1**	**0 (moving frame from 7)**	0.877
3	2 Rtx_2_	0.524	**0**	**1 (ack)_1_**–7 ends	0.913
7	2 Rtx_2_	0.535	**2**	**1 Rtx moving 3**_2_	0.929
**2**	**4(ack)**	0.547	**1**	**2 (ack)**	0.949
**2**	**1 (moving frame from 4)**	0.581	**1**	**0 (moving frame from 3)**	0.979
**1**	**0 (moving frame from 5)**	0.589	**0**	**1 (ack)_1_**–3 ends	79.013

**Table 5.** Average set-up time. BO = 9.

**BO = 9**			

**4 Nodes**		**6 Nodes**	

**SD/BO**	**Time (s)**	**SD/BO**	**Time (s)**
0.25	39.3017952	0.25	44.121011
0.5	36.0465694	0.5	24.4939538
1	43.288385	1	90.7865608

**Table 6.** Standard deviation of the set-up time samples. BO = 9.

**BO = 9**			

**4 Nodes**		**6 Nodes**	

**SD/BO**	**Time (s)**	**SD/BO**	**Time(s)**
0.25	15.3270257	0.25	18.3955348
0.5	23.2507967	0.5	9.54237951
1	43.288385	1	11.99234

**Table 7.** Average set-up time. BO = 7.

**BO = 7**			

**4 Nodes**		**6 Nodes**	

**SD/BO**	**Time (s)**	**SD/BO**	**Time(s)**
0.25	38.3346878	025	28.8846584
0.5	48.6352684	0.5	42.6441296
1	15.9716565	1	18.850547

**Table 8.** Standard deviation of the set-up time samples. BO = 7.

**BO = 7**			

**4 Nodes**		**6 Nodes**	

**SD/BO**	**Time (s)**	**SD/BO**	**Time (s)**
025	32.184823	0.25	17.8481252
0.5	22.9810905	0.5	32.1651341
1	15.9716565	1	3.10913004

**Table 9.** Percentage of packets that received ACKs on the first attempt for BO = 9.

**End Device**		**ECG**	

**SD/BO**	**Percentages**	**SD/BO**	**Percentages**
0.25	54.26%	0.25	38.88%
0.5	41.36%	0.5	60.02%
1	52.45%	1	50.86%

**Table 10.** Percentage of Packages that received ACK on the first attempt BO = 7.

**End Device**		**ECG**	

**SD/BO**	**Percentages**	**SD/BO**	**Percentages**
0.25	44.39%	0.25	50.02%
0.5	58.70%	0.5	67.57%
1	45.13%	1	51.46%
